# Physical fitness, physical activity and adiposity: associations with risk factors for cardiometabolic disease and cognitive function across adolescence

**DOI:** 10.1186/s12887-022-03118-3

**Published:** 2022-02-02

**Authors:** Ryan A. Williams, Simon B. Cooper, Karah J. Dring, Lorna Hatch, John G. Morris, Feng-Hua Sun, Mary E. Nevill

**Affiliations:** 1 Exercise and Health Research Group Sport Health and Performance Enhancement (SHAPE) Research Centre Department of Sport Science, Nottingham Trent University, Nottingham, NG11 8NS UK; 2 Department of Health and Physical Education, The Education University of Hong Kong, Hong Kong SAR, China

**Keywords:** Physical activity, Accelerometery, Cardiometabolic health, Cognitive function, Adolescents, Physical fitness, Adiposity

## Abstract

**Background:**

The cross-sectional associations between physical activity, physical fitness and adiposity with risk factors for cardiometabolic disease (particularly novel ones such as inflammatory cytokines) and cognitive function across the period of adolescence are not well understood. Additionally, novel physical activity metrics that summarise activity volume and intensity in a continuous manner have not been investigated in this context. Therefore, this study investigated the cross-sectional associations between physical activity, physical fitness and adiposity with risk factors for cardiometabolic disease and cognitive function. These associations were compared between younger and older adolescents.

**Methods:**

Seventy younger (11-12y, 35 girls) and 43 older (14-15y, 27 girls) adolescents volunteered to take part in the study. Physical fitness (multi-stage fitness test, MSFT) and adiposity (waist circumference) were determined, followed 7d later by resting blood pressure, a fasted blood sample (glucose, plasma insulin, IL6, IL10, IL15 and IL-1β concentrations) and a cognitive function test battery. Habitual physical activity was monitored via hip-worn accelerometers over this 7-d period and the average acceleration (activity volume), and intensity gradient (intensity distribution of activity) were determined.

**Results:**

Average acceleration and intensity gradient were negatively associated with mean arterial blood pressure (β = -0.75 mmHg, *p* = 0.021; β = -10 mmHg, *p* = 0.006, respectively), and waist circumference was positively associated with IL-6 concentration (β = 0.03%, *p* = 0.026), with stronger associations observed in older adolescents. Higher physical fitness (MSFT distance) was positively associated with anti-inflammatory IL-15 concentration (β = 0.03%, *p* = 0.038) and faster response times on the incongruent Stroop task (β = -1.43 ms, *p* = 0.025), the one-item level of the Sternberg paradigm (β = -0.66 ms, *p* = 0.026) and the simple (β = 0.43 ms, *p* = 0.032) and complex (β = -2.43 ms, *p* = 0.020) levels of the visual search test, but these were not moderated by age group.

**Conclusions:**

The present study highlights the important role of physical activity (both the volume and intensity distribution) and physical fitness for cardio-metabolic health. Furthermore, the present study highlights the importance of physical fitness for a variety of cognitive function domains in adolescents, irrespective of age.

## Background

Although young people do not typically present with cardiometabolic diseases such as cardiovascular disease and type 2 diabetes [1], the risk factors for these conditions are present during the early years of life [1, 2] and track into adulthood and influence lifelong health [3]. During adolescence, the body undergoes many developmental changes [4], which can lead to a state of insulin resistance, and is further complicated by excess adiposity during this period [5]. Indeed, the number of adolescents with type 2 diabetes is increasing each year [6]. Thus, adolescence is a crucial time to identify the prevalence of risk factors for cardiometabolic disease, and their associations with related behaviours and characteristics, such as adiposity and physical fitness, which are modifiable. In addition to cardiometabolic health, the importance of cognition during adolescence has been recognised, as it is related to lifelong physical and mental health [7], as well as academic performance [8]. However, despite the importance of cardiometabolic health and cognition during adolescence, the associations between key modifiable lifestyle factors (such as physical activity, physical fitness, and adiposity) and risk factors for cardiometabolic disease and cognitive function during adolescence are relatively unexplored.

The role of device-measured physical activity is of particular interest, given that physical activity is central to UK national [9] and global [10] guidelines for well-being. Whilst it is generally accepted that physical activity has beneficial effects on traditional risk factors for cardiometabolic disease such as blood pressure [11, 12] and insulin sensitivity [12] in boys and girls between 10–19 years of age; much less is known about the impact of physical activity on novel risk factors for cardiometabolic disease. A key novel risk factor for cardiometabolic disease that has been studied more recently is elevated cytokine concentrations, a marker of low-grade chronic inflammation [1, 13]. In the few studies conducted to date, no association was found between physical activity and IL-6 concentration in boys and girls aged 13 – 17 y [14, 15] and no studies have examined other cytokines related to low-grade inflammation (such as IL-1β, IL-10, IL-15). Additionally, the association between habitual physical activity and cognitive function during adolescence is unclear, with some studies suggesting a beneficial association with the domains of inhibitory control and cognitive flexibility in boys and girls [16, 17], whilst others did not find such associations [18]. A recent omnibus review highlighted the benefits of planned/structured physical activity for cognitive function in young people, however the associations between device-measured physical activity and cognitive function were equivocal [19]. To the author’s knowledge, no studies have examined whether age group (i.e. younger, 11–12 years; older, 14–15 years) moderate the associations with risk factors for cardiometabolic disease and cognitive function during adolescence.

One possible explanation for the lack of association between device-measured physical activity and cardiometabolic health, and cognitive function, is the challenge of assessing device-measured physical activity; including the divergent methodologies used and the numerous protocols for processing and categorising accelerometery data [20, 21]. To address this, Rowlands et al. [22] have proposed two new metrics that continuously capture the volume (average acceleration) and intensity (intensity gradient) of physical activity, overcoming some of the limitations of the more traditional cut-point based approaches. Recent studies have shown that these new metrics (both the average acceleration and intensity gradient) are negatively and independently associated with BMI in 9–11 year-old boys and girls [23, 24] and a composite score of metabolic syndrome risk in boys and girls aged 9–10 years [24]. Despite these initial promising findings, there are currently no data in older adolescents (14–18 years) and no data on the associations between these two new physical activity metrics (average acceleration and intensity gradient physical activity), and traditional metabolic risk factors such as insulin sensitivity. Furthermore, no studies have examined the associations between the new physical activity metrics and inflammatory risk factors for cardiometabolic disease (such as low-grade chronic inflammation), and no studies have examined the associations between the novel physical activity metrics and cognitive function.

There is a strong evidence base that physical fitness is beneficially associated with traditional risk factors for cardiometabolic disease, such as blood lipids [11, 25], HOMA-IR [14, 25, 26] and blood pressure [11] in boys and girls across adolescence. In addition, a small number of previous studies in boys and girls aged 13–14 y [27] and 11–12 y [28] have shown that lower physical fitness is associated with increased pro-inflammatory IL-6 [27, 28] and IL-1β [28] concentrations, as well as a lower anti-inflammatory IL-10 concentration [28]. However, there are no data on how the effect of physical fitness on traditional and novel risk factors for cardiometabolic disease may change across adolescence or if there are relationships between physical fitness and other cytokines which reflect cardiometabolic disease risk. For example, IL-15 is an anti-inflammatory cytokine which is involved in adipose tissue regulation [29] and improved insulin sensitivity [30], yet has not been examined in association with physical fitness.

Physical fitness is also beneficially associated with a range of cognitive function domains in healthy children [16, 31], children living with obesity [32] and adults [33]. Less is known about adolescents, but a small number of studies have shown that physical fitness is positively associated with academic performance [34], inhibitory control [35, 36] and working memory [36] in boys and girls aged 11–15 y. Furthermore, it is interesting that the relationships between device-measured moderate-to-vigorous physical activity (MVPA) and cognitive function found by Aadland et al. [16] no longer remained when physical fitness was considered; suggesting that physical fitness may be more important than habitual physical activity for cognition in adolescents. Thus, it is important to address the possible independent impacts of both physical activity and physical fitness on cognitive function across adolescence.

Adiposity is recognised as an important risk factor for the development of cardiovascular disease and type 2 diabetes [37]. In boys and girls aged 11–12, sum of four skinfolds was positively related to HOMA-IR and mean arterial pressure [28], and in boys and girls aged 13–17 the sum of six skinfolds was positively related with C-reactive protein (CRP) [14]. Adiposity, measured by waist circumference, has also previously been assessed as an outcome related to cardiometabolic disease in adolescents – commonly used in the formation of a composite risk score [11, 12, 25]. There has, however, been less consideration as to how waist circumference influences novel risk factors for cardiometabolic disease in adolescents, such as low-grade chronic inflammation. Furthermore, there is growing evidence to suggest that adiposity may be detrimental for cognitive function, with worse executive function task performance seen in young adults with a higher BMI [38]. On top of this, focus on the association between adiposity and cognitive function has been centred on children [39], with less attention in an adolescent population. Some limited data in adolescents show that boys and girls, aged 13–15, with a higher BMI had worse performance on an attention and cognitive flexibility task [40].

Therefore, the purpose of the present study was to examine the associations between physical activity, physical fitness and adiposity, and risk factors for cardiometabolic disease and cognitive function in a sample of adolescents (age 11–15 years). Furthermore, the present study will also examine if these associations are modified by year group (year 7 (age 11–12 years) and year 10 (age 14–15 years).

## Methods

### Experimental design

The study conformed to the Declaration of Helsinki and was approved by Nottingham Trent University Human Ethics Committee. Informed parental consent was obtained from the parents before enrolment onto the study. A health screen was completed by the parent/guardian on behalf of the participant, which was checked by a lead investigator to ensure there were no medical conditions that would affect the young person’s participation in the study, which included any existing neurological and/or underlying health conditions, such as family history of cardiovascular disease, diabetes, ADHD. All participants enrolled were considered healthy.

The study employed a cross-sectional design, consisting of two main experimental trials that took place at the schools (East Midlands, UK; between May 2018 and January 2020), separated by at least 7 d. In the first experimental trial, participants underwent anthropometric measurements (stature, sitting stature, body mass, waist circumference and skinfolds), as well as completing a 15 m multi-stage fitness test for the assessment of physical fitness. Following the completion of these measures, participants were then familiarised with the battery of cognitive function tests (described below). At the end of the first trial, Actigraph GT3X + accelerometers were given to participants to wear for the 7 d period between the two main trials, for the assessment of free-living physical activity. After the 7 d of activity tracking, participants attended the second main trial following an overnight fast (from 10 pm the previous evening). This trial started with the assessment of blood pressure, followed by a capillary blood sample. Following this, participants were then fed a standardised breakfast providing 1.5 g per kg body mass of carbohydrate [41, 42], before completing the cognitive function test battery.

### Participants

Seventy participants (35 girls) in year 7 and 43 participants (27 girls) in year 10 participated in the study. Stature and sitting stature were measured with a Leicester Height Measure (Seca, Hamburg, Germany) accurate to 0.1 cm and body mass was measured using a Seca 770 digital scale (Seca, Hamburg, Germany) accurate to 0.1 kg. Stature and sitting stature were used to predict age from peak height velocity, following methods described previously [43]. Body mass and stature were used to determine body mass index (BMI), for which age- and sex-specific centiles were derived based on national reference values [44]. Skinfolds were taken at four sites (triceps, subscapular, supraspinale and front thigh), using methods previously described [28, 36]; with the sum of skinfolds used as the criterion measure. All descriptive characteristics of the participants can be seen in Table 1.

**Table 1:** Participant characteristics presented overall, and by sex, for both year groups. Data are presented as mean ± SD

Characteristic	Overall		Boys		Girls	
	Year 7 (*n* = 70)	Year 10 (*n* = 43)	Year 7 (*n* = 35)	Year 10 (*n* = 16)	Year 7 (*n* = 35)	Year 10 (*n* = 27)
Age (y)	11.4 ± 0.5	14.3 ± 0.5	11.4 ± 0.5	14.3 ± 0.4	11.3 ± 0.5	14.3 ± 0.5
Height (cm)	154.5 ± 7.9	169.0 ± 6.3	155.3 ± 8.7	174.1 ± 5.2	153.6 ± 6.9	166.1 ± 4.8
Body mass (kg)	45.8 ± 9.3	60.0 ± 9.6	45.6 ± 9.7	62.0 ± 10.1	46.0 ± 9.0	58.1 ± 9.2
BMI Percentile	63.9 ± 28.1	61.4 ± 29.4	64.3 ± 28.5	64.3 ± 29.5	63.5 ± 28.1	59.7 ± 29.7
APHV (y)	-1.0 ± 0.8	1.9 ± 0.8	-1.6 ± 0.5	1.1 ± 0.4	-0.4 ± 0.6	2.3 ± 0.5
Sum of skinfolds (mm)	59.0 ± 25.7	60.6 ± 26.2	55.5 ± 24.3	49.3 ± 24.6	62.0 ± 26.1	66.4 ± 25.9

*BMI*, body mass index *APHV*, Age from peak height velocity *MSFT*, multi-stage fitness test

### Measurements

#### Physical fitness

Participants completed the 15 m version of the multi-stage fitness test (MSFT), which is a commonly used field-based measurement of physical fitness in youth [45]. The 15 m version starts at 6 km·h^−1^ and increases by 0.5 km·h^−1^ for every subsequent stage (approximately 1 min per stage). Participants were fitted with a heart rate monitor (First Beat Technologies Ltd., Finland) prior to the start and heart rate was recorded continuously during the MSFT. Participants were paced by an experienced member of the research team and were instructed to run until volitional exhaustion; verbal encouragement was provided. The total distance completed (m) was used as the performance criterion for the test.

Waist circumference.

Waist circumference was measured at the narrowest abdominal point, between the lower margin of the lowest palpable rib and the iliac crest, to the nearest 0.1 cm [46]. Waist circumference was used as the chosen surrogate of adiposity [47].

#### Blood pressure

Participants were seated quietly for 5 min prior to the measurement of blood pressure. Blood pressure was measured twice on the right arm, which was rested at chest height, in accordance with guidelines [48], using an automated sphygmomanometer (HBP-1300, Omron, Milton Keynes, UK). If systolic blood pressure differed by > 5 mmHg then a third measurement was taken. All blood pressure readings were interspersed by 1 min rest. The average was used if two measures were conducted, whereas the median was selected if a third measurement was required. Mean arterial blood pressure was determined using the following calculation: diastolic blood pressure + ([0.33*(systolic blood pressure – diastolic blood pressure]) [49].

#### Capillary blood sample

In order to increase capillary blood flow, participants’ hands were warmed via submersion in warm water prior to collection. A unistik single-use lancet (Unistik, Extra, 21G gauge, 2.0 mm depth, Owen Mumford Ltd, UK) was used and the blood collected into a 300 μl EDTA coated microvette (Sarstedt Ltd, UK) and a 300 μl clotting activator coated microvette (Sarstedt Ltd, UK). A single 25 μl whole blood sample was also collected, using a pre-calibrated glass pipette (Hawksley Ltd, UK) and immediately deproteinised in 250 μl ice-cooled 2.5% perchloric acid in 1.5 ml plastic vials. The whole blood and EDTA coated microvettes were centrifuged at 4000 × g, for 4 min at 4 °C (Eppendorph 541C, Hamburg, Germany). The microvette with clotting activator was allowed to rest at room temperature for 30 min before centrifugation at 1000 × g for 15 min. Plasma and serum were extracted into 500 μl vials for subsequent analysis. All samples were frozen immediately at -20 °C and transferred to a -80 °C freezer as soon as possible.

#### Blood sample analysis

Blood glucose concentrations were measured in duplicate (GOD/PAP method, GL364, Randox, Ireland) and plasma insulin concentrations were measured in singular (ELISA; Mercodia Ltd, Sweden), using commercially available methods and according to the manufacturer’s instructions. Fasting blood glucose and plasma insulin concentrations were used to calculate the homeostatic model assessment of insulin resistance (HOMA-IR) [50]. The intra-assay coefficients of variation (%) for 8 repeat measurements were; blood glucose (3.8%) and plasma insulin (4.4%).

Plasma concentrations of IL-6, IL-10, IL-15 and IL-1β were determined using the Ella SimplePlex automated immunoassay (ProteinSimple, BioTechne, Oxford, UK), in line with the manufacturer’s instructions [51]. Samples and wash buffer were added to cartridge inlets, before being loaded into the machine. Briefly, sample is routed through separate microfluidic channels which are coated with analyte-specific antibodies. The channels are washed and then a detection antibody is applied. The detection antibody migrates through the microfluidic channel, into the Glass Nano Reactors (GNRs) whereby the sample is measured in triplicate. Concentrations are generated from the factory-calibrated standard curves that are preloaded into the cartridge. The microfluidic technology used by the Ella system allows for the simultaneous quantification of biomarkers without cross-reactivity [51] and cytokine concentrations using this method have been strongly correlated with ELISA-based concentrations [51]. The intra-assay coefficients of variation (%) for 8 repeat measurements were; IL-6 (3.8%), IL-10 (3.9%), IL-15 (2.9%) and IL-1β (2.5%).

Brain-derived neurotrophic factor (BDNF) concentrations were determined with a commercially available ELISA (Quantikine ELISA ®, R & D Systems Europe Ltd, UK) according to the manufacturer’s instructions. The intra-assay coefficient of variation (%) for 8 repeat measurements was 7.3%.

#### Cognitive function tests

The cognitive function test battery lasted approximately 15 min and consisted of the Visual Search Test (assessing visual perception), Stroop test (assessing executive function), Sternberg Paradigm (assessing working memory) and a Flanker task (assessing executive function) which were completed on a laptop computer (Lenovo ThinkPad T450; Lenovo, Hong Kong), as previously described [41, 42]. Instructions for each test were presented prior to completion of the test and participants had an opportunity to ask questions before starting. Each test, and test level, were preceded by practice stimuli in order to re-familiarise participants with the test and negate any potential learning effects; the data for the practice stimuli were discarded. The participants completed the tests in a classroom, in silence and separated so that they could not interact during the tests. Sound cancelling headphones were also worn, to minimise external disturbances. The variables of interest from each test were the response times of correct responses and the proportion (%) of correct responses. This testing procedure has been used successfully in a similar study population previously [36, 52].

#### Assessment of physical activity

Free-living, device-measured physical activity was assessed with ActiGraph GT3X + triaxial accelerometers (Actigraph, Pensacola, FL, USA). Participants were instructed to wear the accelerometer at all times (24 h^.^d^−1^), with the monitor positioned on an elasticated waist band just above the right hip, for seven consecutive days. Participants were asked only to remove the accelerometer for any water-based activities, such as swimming or showering. To ensure that the accelerometer was correctly placed, participants were fitted with the accelerometer by a member of the research team. The accelerometers were initialised at a sampling rate of 90 Hz.

Processing of accelerometer data.

Data were downloaded using Actilife (v6.13.4; Actigraph, Pensacola, FL, USA) and saved in raw format (.gt3x files), before conversion to raw (.csv) file format for raw signal processing. The raw files were processed using RStudio (v1.2.1335; R Studio Team, 2020) using the GGIR v2.0–0 package (for a detailed explanation on the steps involved, please see Migueles et al. [53]). Briefly, GGIR auto calibrates the file and identifies non-wear time [54]. The default setting for non-wear was used, whereby non-wear data was imputed by the average at similar time points on the other days of the week [54]. As a result, all accelerometery outcomes are based on complete 24 h cycles (1440 min). The raw triaxial acceleration signals are then converted into a single, omnidirectional measure of acceleration accounting for gravity (1 g), termed the Euclidean Norm Minus One (ENMO) [54]. The ENMO is computed over 5 s epochs and expressed in milli-gravitational (*mg*) units, as previously described [22]. Participants’ accelerometery data were excluded if there was a post-calibration error greater than 0.01 g, there was less than 3 valid days (including 1 weekend day) of wear time (defined as ≥ 16 h per day), or data was not present for full 24 h wear cycles [22]. A total of 20 accelerometery files were excluded from analysis (absent on data collection, *n* = 1; insufficient wear time, *n* = 16; calibration error > 0.01 g, *n* = 3).

Based on the descriptions provided by Rowlands et al. [22], the total volume of physical activity per day was expressed as the average acceleration (ENMO, *mg*). The intensity gradient was the metric of choice to describe the intensity of physical activity. For detailed information on the inception, and description, of the intensity gradient see Rowlands et al. [22]. Briefly, the intensity gradient describes the relationship between the log values of intensity (represented by intensity bins of 25 mg resolution, i.e. 0 – 25 mg, 25 – 50 mg…3975 – 4000 mg) and time (accumulated time in each intensity bin). The intensity gradient is always negative, as this reflects the decrease in time spent in higher intensity activities. The average intensity gradient over a 24 h period was calculated for each participant, as well as the constant for the linear regression equation and the *R*^*2*^ value (indicative of the goodness of fit for the model). In brief, a less negative intensity means more time is spent doing higher intensity activities whereas a more negative intensity gradient means less time is spent doing higher intensity activities.

### Statistical analysis

All analyses were performed using RStudio (RStudio Team, 2020). Multiple linear regression was performed, using the “lme4” package [55], to examine associations between exposure variables; physical fitness (distance run on the MSFT), physical activity (average acceleration and intensity gradient) and adiposity (waist circumference) with outcome variables: risk factors for cardiometabolic disease and cognitive function. The models were first performed whilst adjusting for year group and sex, to determine the overall association of the exposure variable of interest. Following this, an interaction term consisting of year group and the exposure variable of interest (physical fitness, average acceleration, intensity gradient or adiposity) was included, to determine a moderating effect of year group on the relationship. All exposure variables were centred before entry into the models and residuals were assessed for conformity with the underlying assumptions of normality and homoscedasticity. Variance inflation factors (VIF) were used to assess collinearity (all VIF’s were < 5) and therefore satisfied this assumption [56]. Residual analyses were performed and if normality or homoscedasticity were violated, the dependent variable was log transformed and the residuals were checked thereafter. For models where the log version was used, the coefficients and 95% CI are presented as a % change for a 1-unit increase in the exposure variable. For all analyses, alpha for determining statistical significance was set at *p* < 0.05.

## Results

Descriptive summaries, split by year group and sex, are presented below: exposure variables—physical activity, physical fitness and adiposity (Table 2); risk factors for cardiometabolic disease (Table 3); cognitive function outcomes and BDNF concentration (Table 4).

**Table 2:** Descriptive summary of physical activity, physical fitness and adiposity measurements, split by year group and sex. Data are mean ± SD and (range)

Variable	Year 7		Year 10	
	*Boys*	*Girls*	*Boys*	*Girls*
*Average Acceleration (mg)*	17.7 ± 4.6 (11.4 – 29.6)	13.3 ± 3.4 (5.8 – 20.1)	14.2 ± 4 (7.5 – 20.7)	12.5 ± 3.4 (8.1 – 21.9)
*Intensity Gradient (AU)*	-2.34 ± 0.14 (-2.58 – -2.12)	-2.51 ± 0.19 (-2.83 – -2.13)	-2.43 ± 0.23 (-2.87 – -1.98)	-2.57 ± 0.19 (-2.86 – -2.23)
*MSFT (m)*	1395 ± 435 (720–2250)	1215 ± 435 (540–2550)	2295 ± 450 (1650–3240)	1380 ± 405 (720–2460)
*Waist Circumference (cm)*	65.8 ± 6.9 (56.7 – 84.0)	66.5 ± 7.4 (55.9 – 84.1)	72.8 ± 6.4 (62.8 – 91.6)	68.2 ± 6.4 (60.5 – 91.0)

*MSFT*, Multi-Stage Fitness Test

Data are mean ± SD and (range)

**Table 3:** A descriptive summary of the risk factors for cardiometabolic disease, split by year group and sex. Data are mean ± SD and (range)

Variable	Year 7		Year 10	
	**Boys**	**Girls**	**Boys**	**Girls**
Systolic Blood Pressure (mmHg)	115 ± 12 (95 – 146)	115 ± 11 (96 – 139)	128 ± 13 (107 – 153)	124 ± 8 (105 – 146)
Diastolic Blood Pressure (mmHg)	69 ± 7 (59 – 84)	70 ± 7 (58 – 86)	73 ± 8 (56 – 85)	77 ± 6 (63 – 87)
Mean Arterial Pressure (mmHg)	84 ± 7 (72 – 99)	84 ± 7 (71 – 100)	91 ± 9 (72 – 107)	92 ± 6 (80 – 101)
Blood Glucose Concentration (mmol∙L^−1^)	4.02 ± 0.45 (3.10 – 5.0)	4.12 ± 0.59 (3.30 – 5.80)	4.43 ± 0.75 (3.0 – 5.50)	4.52 ± 0.53 (3.60 – 5.50)
Plasma Insulin Concentration (pmol∙L^−1^)	54.5 ± 32.3 (14.7 – 123.0)	67.7 ± 29.9 (22.8 – 123.0)	51.2 ± 25.8 (13.3 – 96.7)	60.2 ± 25.8 13.3 – 113.0)
HOMA-IR (AU)	1.58 ± 0.91 (0.39 – 3.40)	2.09 ± 1.02 (0.55 – 4.38)	1.65 ± 0.99 (0.39 – 3.53)	2.00 ± 0.85 (0.35 – 3.82)
IL-6 Concentration (pg∙ml^−1^)	1.70 ± 1.06 (0.53 – 4.68)	2.59 ± 1.33 (0.36 – 5.34)	1.38 ± 0.73 (0.59 – 3.39)	1.17 ± 0.55 (0.44 – 2.33)
IL-10 Concentration (pg∙ml^−1^)	2.97 ± 0.90 (0.69–5.79)	3.88 ± 1.70 (0.97 – 8.27)	2.81 ± 0.59 (1.70 – 3.82)	2.65 ± 1.08 (1.25 – 6.09)
IL-15 Concentration (pg∙ml^−1^)	2.45 ± 0.63 (1.28 – 3.85)	3.44 ± 1.11 (1.39 – 5.87)	2.94 ± 0.85 (1.78 – 5.4)	3.17 ± 1.17 (1.26 – 5.90)
IL-1β Concentration (pg∙ml^−1^)	41.0 ± 25.5 (3.55 – 106.00)	38.4 ± 20.2 (7.37 – 74.80)	43.5 ± 23.6 (6.97 – 80.60)	30.2 ± 16.9 (5.65– 76.60)

*HOMA-IR*, Homeostatic Model Assessment of Insulin Resistance IL, Interleukin

**Table 4:** A descriptive summary of cognitive function outcomes and BDNF concentration, split by year group and sex. Data are mean ± SD (range)

Test	Level	Variable	Year 7		Year 10	
			*Boys*	*Girls*	*Boys*	*Girls*
Stroop	Congruent	RT (ms)	856 ± 118 (652—1116)	818 ± 141 (611–1211)	644 ± 68 (515–780)	663 ± 81 (516–846)
		Accuracy (%)	96.0 ± 5.2 (75.0 – 100)	97.2 ± 3.9 (85.0 – 100)	97.5 ± 4.10(85.0 – 100)	96.3 ± 4.4(85.0 – 100)
	Incongruent	RT (ms)	1245 ± 229(905 – 1788)	1135 ± 216(799 – 1572)	837 ± 114(658 – 1010)	942 ± 171(680 – 1340)
		Accuracy (%)	89.2 ± 7.8(62.5 – 97.5)	93.1 ± 6.3(77.5 – 100)	93.9 ± 4.9 (80.0 – 100)	93.9 ± 7.5 (62.5 – 100)
Flanker	Congruent	RT (ms)	610 ± 87 (448–801)	631 ± 165 (414–1171)	507 ± 69 (417–687)	491 ± 77 (365–738)
		Accuracy (%)	96.9 ± 5.8 (76.7 – 100)	96.5 ± 7.3 (60.0 – 100)	98.1 ± 2.7 (93.3 – 100)	97.8 ± 3.3 (86.7 – 100)
	Incongruent	RT (ms)	666 ± 107 (468 – 906)	682 ± 185 476 – 1176)	550 ± 57 (461 – 709)	534 ± 88 (406 – 857)
		Accuracy (%)	90.2 ± 14.9 (40.0 – 100)	89.6 ± 16.3 (36.7 – 100)	95.2 ± 4.7 (80.0 – 100)	95.5 ± 3.8 (83.3 – 100)
Sternberg paradigm	One item	RT (ms)	577 ± 93 (412–826)	563 ± 117 (408–896)	465 ± 62 (364–568)	468 ± 80 (363–730)
		Accuracy (%)	91.7 ± 12.6 (50.0 – 100)	95.2 ± 7.1 (75.0 – 100)	94.1 ± 6.2 (75.0 – 100)	96.2 ± 6.8 (75.0 – 100)
	Three item	RT (ms)	750 ± 160 (377 – 1110)	692 ± 172 (397 – 1257)	556 ± 62 (463 – 662)	546 ± 87 (294 – 767)
		Accuracy (%)	92.8 ± 10.4 (43.8 – 100)	91.5 ± 15.5 (12.5 – 100)	95.5 ± 3.6 (87.5 – 100)	92.4 ± 18.9 (3.1 – 100)
	Five item	RT (ms)	920 ± 171 (445 – 1477)	873 ± 182 (574 – 1594)	677 ± 88 (557 – 882)	677 ± 113 (518 – 130)
		Accuracy (%)	86.7 ± 11.5 (50.0 – 100)	90.7 ± 8.4 (62.5 – 100)	92.8 ± 6.4 (81.2 – 100)	93.9 ± 5.9 (81.2 – 100)
Visual search	Simple	RT (ms)	592 ± 83 (474 – 819)	579 ± 70 (483 – 830)	512 ± 33 (471 – 568)	536 ± 37 (482 – 605)
		Accuracy (%)	92.3 ± 10.9 (47.7 – 100)	95.4 ± 6.9 (65.6 – 100)	96.8 ± 4.6 (84.0 – 100)	97.8 ± 3.3 (91.3 – 100)
	Complex	RT (ms)	1573 ± 408 (777 – 2726)	1577 ± 546 (938 – 3130)	1375 ± 244 (927 – 1936)	1348 ± 324 (910 – 2132)
		Accuracy (%)	87.2 ± 22.6 (10.1 – 100)	90.1 ± 18.0 (26.9 – 100)	98.7 ± 4.1 (26.9 – 100)	98.8 ± 3.1 (87.5 – 100)
BDNF Concentration (ng·ml^−1^)			30.9 ± 7.3 (12.6 – 45.6)	36.2 ± 11.1 (11.8 – 58.5)	32.4 ± 8.8 (16.8 – 42.6)	32.7 ± 10.2 (13.3 – 47.9)

*RT*, Response Time *VST*, Visual Search Test *BDNF*, Brain-Derived Neurotrophic Factor

Data are mean ± SD and (range)

### Associations between physical activity, physical fitness and adiposity with risk factors for cardiometabolic disease

The cross-sectional associations, adjusted for year group and sex, between exposure variables: physical fitness (distance covered on the MSFT), physical activity (average acceleration and intensity gradient) and adiposity (waist circumference) with outcome variables: risk factors for cardiometabolic disease are presented in Table 5.

**Table 5:** Cross-sectional associations between physical fitness, physical activity and waist circumference and risk factors of cardiometabolic disease, when controlling for year group and sex. Coefficients (β) are presented in their unstandardised forms, unless otherwise stated, with 95% confidence intervals

Dependent Variables	MSFT		Average Acceleration		Intensity Gradient		Waist Circumference	
	*β (95% CI)*	*p*	*β (95% CI)*	*p*	*β (95% CI)*	*p*	*β (95% CI)*	*p*
*Systolic Blood Pressure (mmHg)*	-0.004 (-0.008, 0.008)	0.106	**-0.83 (-1.39, -0.29)**	**0.005**	**-19.10 (-30.68, -7.44)**	**0.002**	**0.32 (0.02, 0.61)**	**0.038**
*Diastolic Blood Pressure (mmHg)*	-0.002 (-0.004, 0.0003)	0.222	**-0.41 (-0.77, -0.04)**	**0.031**	-6.84 (-14.50, 0.83)	0.084	-0.02 (-0.20, 0.15)	0.815
*Mean Arterial Pressure (mmHg)*	-0.03 (-0.06, -0.001)	0.113	**-0.55 (-0.90, -0.19)**	**0.004**	**-10.92 (-18.45, -3.40)**	**0.006**	0.09 (-0.10, 0.27)	0.377
*Blood Glucose (mmol·L*^*−1*^*)*	-0.0015 (-0.004, 0.001)	0.118	-0.01 (-0.04, 0.02)	0.488	-0.10 (-0.74, 0.56)	0.785	0.01 (-0.01, 0.02)	0.649
*Plasma Insulin (pmol·L*^*−1*^*)*	-0.003 (-0.18, 0.17)	0.971	0.05 (-1.56, 1.66)	0.951	-24.70 (-57.70, 8.28)	0.146	0.70 (-0.19, 1.59)	0.125
*HOMA-IR (AU)*	-0.002 (-0.007, 0.004)	0.484	-0.02 (-0.07, 0.04)	0.555	-0.93 (-1.99, 0.13)	0.091	0.02 (-0.01, 0.05)	0.115
*IL-6*^*a*^	0.15 (-0.14, 0.44)	0.367	-0.60 (-11.63, 11.81)	0.712	-6.20 (-50.97, 79.46)	0.846	1.31 (-0.27, 2.91)	0.093
*IL-10*^*a*^	0.09 (-0.15, 0.33)	0.462	0.40 (-1.75, 2.59)	0.711	16.42 (-26.26, 83.80)	0.515	0.80 (-0.18, 1.80)	0.162
*IL-15*^*a*^	**0.30 (0.07, 0.54)**	**0.038**	-0.70 (-2.45, 1.09)	0.421	17.35 (-18.98, 69.97)	0.402	-0.40 (-1.37, 0.58)	0.359
*IL-1β*^*a*^	0.45 (-0.14, 1.04)	0.061	2.33 (-1.80, 6.63)	0.280	12.64 (-51.13, 159.60)	0.780	0.60 (-1.39, 2.63)	0.557

*MSFT*, Multi-Stage Fitness Test *CI*, Confidence Interval *HOMA-IR*, Homeostatic Model Assessment of Insulin Resistance *IL*, Interleukin

Coefficient and CI are presented as a 1-unit change in the dependent variable for a 1-unit change in MSFT (15 m), average acceleration (1 mg), intensity gradient (1 AU), waist circumference (1 cm)

^a^dependent variable is log transformed. For log transformed data, the coefficient and CI are presented as the % change for a 1-unit change in the predictor variables (MSFT, average acceleration, intensity gradient and waist circumference)

Significant associations are highlighted in **bold** (*p* < 0.05)

**Fig. 1:**
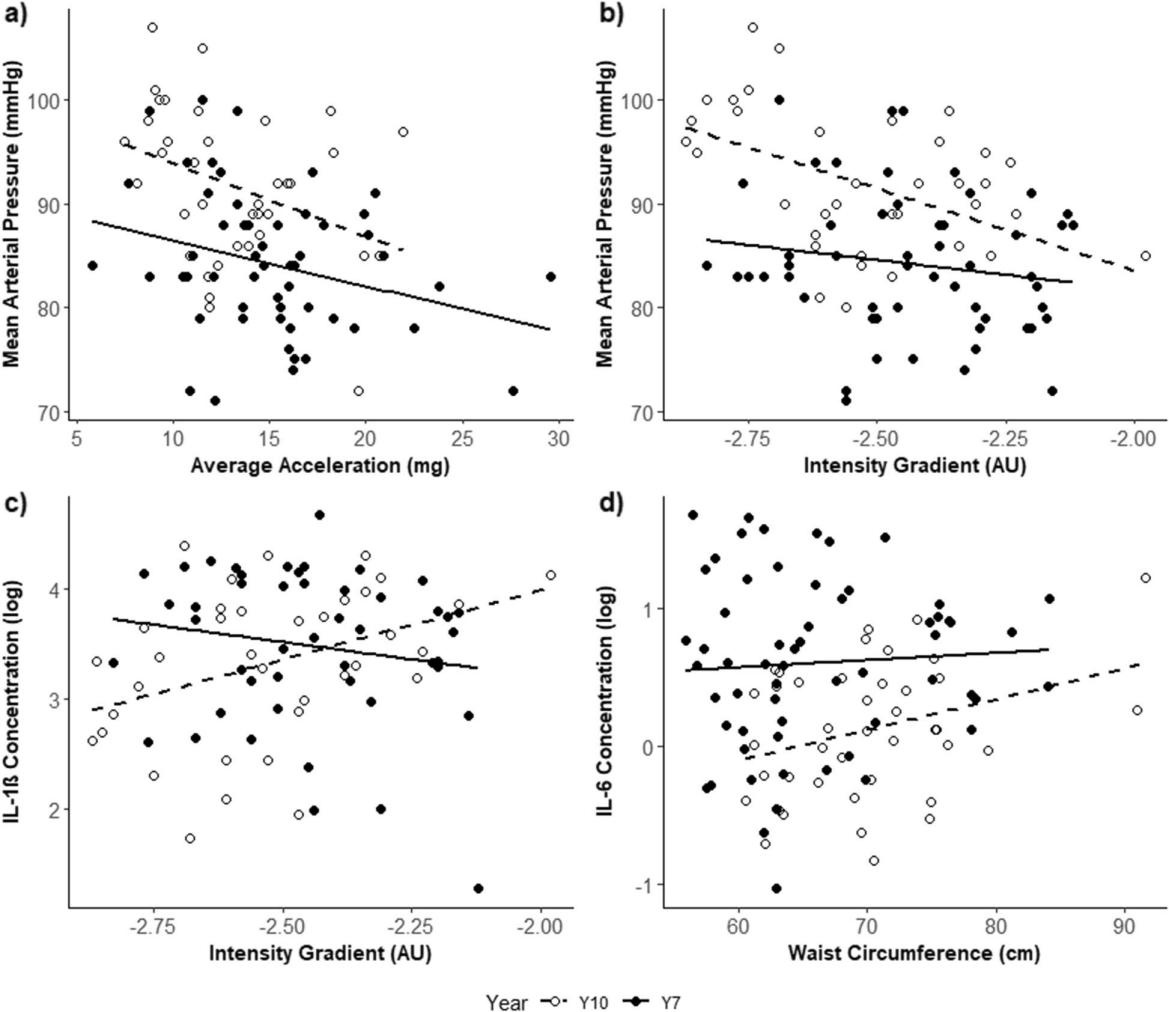
Cross-sectional associations between physical activity metrics (average acceleration and intensity gradient) and waist circumference with blood pressure, as well as IL-6 and IL-1β concentrations. Separate lines are fit to show the year-group specific relationships. (**a**) Association between average acceleration (mg) and mean arterial pressure (mmHg) for year 10 (open points: β = -0.71 mmHg, *p* = 0.021) and year 7 (solid points). (**b**) Association between intensity gradient (AU) and mean arterial pressure (mmHg) for year 10 (open points: β = -16 mmHg, *p* = 0.003) and year 7 (solid points). (**c**) Association between intensity gradient (AU) and log-transformed IL-1β concentration for year 10 (open points) and year 7 (solid points: β = -86.5%, *p* = 0.010). (**d**) Association between waist circumference (cm) and log-transformed IL-6 concentration for year 10 (solid points: β = 0.03%, *p* = 0.026) and year 7 (open points)

Average acceleration was negatively associated with systolic, diastolic, and mean arterial blood pressure (Table 5). Specifically, a 1 mg higher average acceleration was associated with lower blood pressure; 0.8 mmHg (systolic), 0.4 mmHg (diastolic) and 0.5 mmHg (MAP). Including the interaction term provided evidence that the association was modified by year group for diastolic blood pressure and mean arterial pressure. Specifically, the association with diastolic blood pressure (β = -0.653, *p* = 0.036) and MAP (β = -0.710, *p* = 0.021, Fig. 1A) was stronger in year 10 participants than in year 7 participants. There were no clear associations between average acceleration and the remaining risk factors for cardiometabolic disease (Table 5), nor were these associations moderated by year group (Year group by average acceleration interaction; all *p* > 0.05).

Intensity gradient was negatively associated with systolic and mean arterial blood pressure (Table 5). Specifically, a 1 AU higher intensity gradient was associated with lower blood pressure; 19 mmHg (systolic) and 10 mmHg (MAP). Including the interaction term provided evidence that the associations with systolic, diastolic and mean arterial blood pressure were modified by year group, whereby the association was stronger in year 10 participants (systolic: β = -20.74, *p* = 0.013; diastolic: β = -13.37, *p* = 0.013; MAP: β = -16.03, *p* = 0.003; Fig. 1B). There were no clear associations between intensity gradient and the remaining risk factors for cardiometabolic disease (Table 5), nor were any of these associations moderated by year group (Year group*intensity gradient interaction; all *p* > 0.05), with the exception of IL-1β concentration. The association between intensity gradient and IL-1β concentration was dependent on year group (Year group*Intensity gradient interaction; β = -2 (-86.5%), *p* = 0.010). Specifically, IL-1β concentration was negatively associated with intensity gradient for year 7 participants, whereas it was positively associated in year 10 participants (Fig. 1C).

Physical fitness was positively associated with IL-15 concentration, whereby a 15 m (1 shuttle) higher MSFT performance was associated with a 0.3% higher IL-15 concentration (Table 5). There was no moderating effect of year group on this relationship (Year group*MSFT interaction; *p* = 0.478). There were no clear associations between physical fitness and the remaining risk factors for cardiometabolic disease (Table 4), nor were these associations moderated by year group (Year group*MSFT interaction; all *p* > 0.05).

**Fig. 2:**
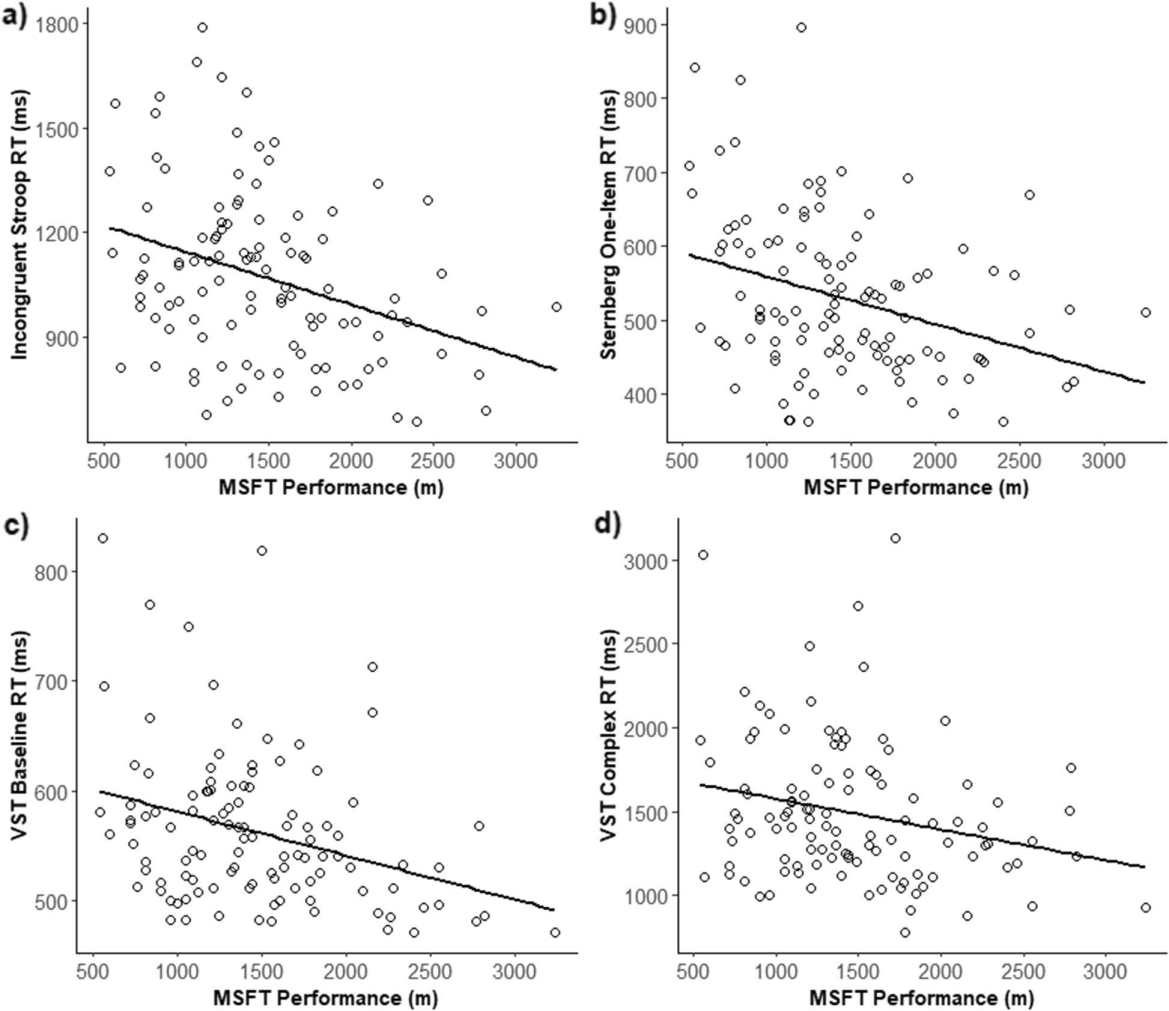
Cross-sectional associations between physical fitness (performance on the multi-stage fitness test) and response times on select cognitive function tasks. (**a**) Association between MSFT performance (m) and response times (RT) on the Incongruent Stroop task (β = -1.43 ms, *p* = 0.025). (**b**) Association between MSFT performance (m) and response times on the One-item Sternberg task (β = -0.66 ms, *p* = 0.036). (**c**) Association between MSFT performance (m) and response times on the baseline level of the Visual Search Test (VST) (β = -0.43 ms, *p* = 0.032). (**d**) Association between MSFT performance (m) and response times on the complex level of the Visual Search Test (β = -2.43, *p* = 0.020)

Waist circumference was positively associated with systolic blood pressure (Table 5). Specifically, a 1 cm higher waist circumference was associated with a 0.3 mmHg higher systolic blood pressure. There were no clear associations between waist circumference and any of the remaining risk factors for cardiometabolic diseases (Table 4), nor were any of these associations moderated by year group (Year group*waist circumference interaction; all *p* > 0.05); with the exception of IL-6 concentration. For year 10 participants, waist circumference was positively associated with IL-6 concentration (β = 0.03% *p* = 0.026), whereas there was no clear evidence of an association for year 7 participants (*p* = 0.123, Fig. 2).

### Associations between physical fitness, physical activity and adiposity with cognitive function

The cross-sectional associations, adjusted for year group and sex, between exposure variables: physical fitness (distance covered on the MSFT), physical activity (average acceleration and intensity gradient) and adiposity (waist circumference) with outcomes: cognitive function (response times) are presented in Table 6.

**Table 6:** Cross–sectional associations with cognitive function outcomes and BDNF concentration, when controlling for year group and sex. Coefficients (β) are presented in their unstandardised forms, unless otherwise stated, with 95% confidence intervals

Test	Level	Variable	MSFT		Average Acceleration		Intensity Gradient		Waist Circumference	
			*β (95% CI)*	*p*	*β (95% CI)*	*p*	*β (95% CI)*	*p*	*β (95% CI)*	*p*
Stroop	Congruent	RT (ms)	-0.360 (-1.04, 0.32)	0.303	-2.69(-8.62, 3.23)	0.375	-60.4 (-183.9, 63.2)	0.341	2.1 (-0.9, 5.1)	0.185
		Accuracy (%)	**0.035 (0.008, 0.062)**	**0.011**	-0.08 (-0.28, 0.12)	0.441	2.14 (-2.17, 6.45)	0.333	-0.47 (-0.59, -0.35)	0.445
	Incongruent	RT (ms)	**-1.43 (-2.66, -0.19)**	**0.025**	-3.92 (-14.30, 6.45)	0.461	-100.5 (-316.7, 115.8)	0.365	2.6 (-2.9, 8.1)	0.356
		Accuracy (%)	**0.045 (0.016, 0.074)**	**0.011**	-0.29 (-0.49, -0.09)	0.092	-0.78 (-7.84, 6.28)	0.831	-0.15 (-0.33, 0.03)	0.108
Flanker	Congruent	RT (ms)	-0.015^a^ (-0.133, 0.103)	0.828	0.17 (-0.77, 1.12)	0.721	0.90^a^ (-17.4, 23.2)	0.928	0.37^a^ (-0.02, 0.77)	0.121
		Accuracy (%)	-0.015 (-0.044, 0.014)	0.952	-0.23 (-0.43, -0.03)	0.121	-1.77 (-8.04, 4.50)	0.578	-0.07 (-0.21, 0.07)	0.326
	Incongruent	RT (ms)	-0.015 (-0.132, 0.103)	0.834	-0.11 (-1.05, 0.83)	0.813	-1.10^a^ (-18.9, 20.6)	0.910	0.40^a^ (-0.10, 0.90)	0.109
		Accuracy (%)	0.003 (-0.073, 0.079)	0.944	0.01 (-0.58, 0.60)	0.960	4.18 (-9.54, 17.90)	0.553	-0.10 (-0.43, 0.23)	0.294
Sternberg paradigm	One item	RT (ms)	**-0.66 (-1.22, -0.10)**	**0.026**	-0.46^a^ (-1.39, 0.48)	0.338	-9.8^a^ (-25.8, 9.8)	0.305	0.40^a^ (-0.05, 0.85)	0.066
		Accuracy (%)	0.015 (-0.038, 0.068)	0.581	-0.31 (-0.80, 0.18)	0.210	-7.04 (-17.23, 3.15)	0.185	0.11 (-0.13, 0.35)	0.337
	Three item	RT (ms)	-0.38 (-1.23, 0.48)	0.387	-3.52 (-10.45, 3.42)	0.323	0.80^a^ (-18.8, 25.1)	0.943	0.21^a^ (-0.34, 0.76)	0.448
		Accuracy (%)	0.051 (-0.037, 0.139)	0.240	-0.30 (-0.89, 0.29)	0.360	3.70 (-10.22, 17.61)	0.605	-0.17 (-0.54, 0.20)	0.359
	Five item	RT (ms)	-0.06^a^ (-0.18, 0.06)	0.246	-0.68^a^ (-1.61, 0.26)	0.160	-13.9^a^ (-29.3, 4.8)	0.138	3.0 (-1.1, 7.1)	0.156
		Accuracy (%)	0.030 (-0.023, 0.083)	0.251	-0.09 (-0.56, 0.38)	0.687	0.81 (-9.18, 10.81)	0.875	0.12 (-0.12, 0.35)	0.311
Visual search	Baseline	RT (ms)	**-0.43 (-0.81, -0.05)**	**0.032**	-0.004 ^a^ (-0.53, 0.53)	0.989	-1.8^a^ (-12.0, 9.6)	0.750	1.10 (-0.68, 2.79)	0.236
		Accuracy (%)	0.007 (-0.038, 0.051)	0.783	0.07 (-0.32, 0.46)	0.717	-2.10 (-10.53, 6.33)	0.626	0.09 (-0.11, 0.29)	0.353
	Complex	RT (ms)	**-2.43 (-4.96, 0.10)**	**0.020**	-0.67^a^ (-2.00, 0.68)	0.334	-0.90^a^ (-25.6, 31.9)	0.953	-0.01^a^ (-0.67, 0.66)	0.982
		Accuracy (%)	-0.068 (-0.156, 0.021)	0.174	-0.65 (-1.43, 0.13)	0.150	-1.30 (-19.72, 17.12)	0.890	0.13 (-0.26, 0.52)	0.554
*BDNF Concentration* (ng*·*ml^−1^)			0.05 (-0.01, 0.11)	0.103	0.14 (-0.41, 0.69)	0.613	5.54 (-5.36, 16.44)	0.321	-0.17 (-0.42, 0.10)	0.210

*MSFT*, Multi-Stage Fitness Test *CI*, Confidence Interval *VST*, Visual Search Test *BDNF*, Brain-Derived Neurotrophic Factor

Coefficient and CI are presented as a 1-unit change in the dependent variable for a 1-unit change in MSFT (15 m), average acceleration (1 mg), intensity gradient (1 AU), waist circumference (1 cm)

For log transformed variables ^a^, the coefficients and CI are presented as a % change in the dependent variable for a 1-unit change in the predictor

Significant associations are highlighted in bold (*p* < 0.05)

For average acceleration, intensity gradient and waist circumference there were no clear associations with performance on any of the cognitive function tests or BDNF concentration (Table 6), nor were any of these relationships moderated by year group (all *p* > 0.05).

Physical fitness was negatively associated with response time on the incongruent Stroop task, whereby a 15 m (1 shuttle) higher MSFT distance was associated with a 1.43 ms faster response time (*p* = 0.025, Fig. 2A). There was no moderating effect of year group on this relationship (Year group*MSFT interaction; *p* = 0.986). Furthermore, physical fitness was positively associated with accuracy on both the congruent (β = 0.04%, *p* = 0.011) and incongruent (β = 0.05%*, p* = 0.011) levels of the Stroop task, although these associations were not moderated by year group (year group*MSFT interactions; congruent; *p* = 0.883. incongruent; *p* = 0.484). Physical fitness was negatively associated with response time on the one-item level of the Sternberg paradigm, whereby a 15 m (1 shuttle) higher MSFT distance was associated with a 0.66 ms faster response time (*p* = 0.036, Fig. 2B). There was no moderating effect of year group on this relationship (Year group*MSFT interaction; *p* = 0.241). Physical fitness was also negatively associated with response times during both levels of the visual search test. Specifically, a 15 m (1 shuttle) higher MSFT distance was associated with a 0.43 ms faster response time on the baseline level (*p* = 0.032) and a 2.43 ms faster response time on the complex level (*p* = 0.020, Fig. 2C & D). There was no moderating effect of year group on these relationships (Year group*MSFT interaction; both *p* > 0.05). For the remaining cognitive function outcomes and BDNF concentration, there were no associations with physical fitness (Table 6), nor were these relationships moderated by year group (Year group*MSFT interaction; all *p* > 0.05).

## Discussion

The main findings of the present study were that: physical fitness (distance covered on the MSFT) was positively associated with anti-inflammatory IL-15 concentration and accuracy on the congruent and incongruent Stroop tasks, and negatively associated with response times across cognitive domains (indicating that higher fit participants displayed faster response times); and that physical activity volume (average acceleration) and physical activity intensity (intensity gradient) were negatively associated with blood pressure (systolic, diastolic and MAP). Furthermore, the present study also demonstrates that boys spent more time in high intensity physical activity compared to girls (as reflected by a less negative intensity gradient value), and that boys and year 10 participants had a higher physical fitness (as measured by distance covered on the MSFT) compared to girls and year 7 participants respectively. Furthermore, the difference in physical fitness between boys and girls was greater in year 10 participants than year 7 participants. In addition, year 7 girls had higher cytokine concentrations (IL-6 and IL-10) in comparison with boys of the same age. Blood pressure and blood glucose concentration were higher in year 10 participants compared to year 7, and year 10 participants were consistently quicker across a range of cognitive function domains (attention, inhibitory control, working memory and visual processing).

A key novel finding of the present study is that physical fitness (distance covered on the MSFT) is positively associated with IL-15 concentration in adolescents. IL-15 has a recognised role in adipose tissue regulation [29] as well as skeletal muscle insulin sensitivity, oxidative metabolism and angiogenesis [30]. Data from animal models suggest that IL-15 activates PPAR-δ which is responsible for a subsequent improvement in endurance capacity through regular training [30]. Our findings extend the previously reported increase in skeletal muscle IL-15 content after 12 weeks of endurance training in adult males [57], by reporting a positive association between physical fitness (MSFT performance) and IL-15 concentration, which may contribute to chronic training adaptations as a result of repeated physical activity. However, longitudinal data following a training intervention in adolescents is required to confirm this suggestion.

Another key finding of the present study was the positive relationships between higher levels of physical fitness and cognitive function in adolescents. Specifically, the present study is also the first to demonstrate that physical fitness is beneficially associated with indices of simple and complex visual processing speed (response times on the visual search test) in adolescents. Higher physical fitness was also associated with faster response times across the domain of executive function (specifically inhibitory control and working memory), which is consistent with much of the evidence base in adolescents [16, 35, 58]. It has been stated that adolescents may be more sensitive to the effects of physical fitness on cognition, particularly with regards to executive function, as the associated brain regions are still developing at this stage [59]. A hypothesised mechanism of these beneficial effects is through the release of neurotrophins, such as brain-derived neurotrophic factor (BDNF) [59]. However, the present study did not support this and found no associations between physical fitness and BDNF concentration. Nonetheless, the data from the present study demonstrate a beneficial association between physical fitness and cognitive function. Future work should seek to explore the potential mechanistic links between physical fitness and cognition, an understanding of which would allow such mechanisms to be targeted in the design of future interventions.

The present study is also the first to demonstrate that the newly proposed physical activity metrics, both the physical activity volume and intensity of the activity, are negatively associated with mean arterial pressure. It is well known that higher physical activity levels are associated with reductions in blood pressure [11, 12] and have protective effects against the development of hypertension [60]. However, the present study provides novel insight across adolescence in that the association for both physical activity variables was dependent on year group, whereby a stronger negative association was observed in year 10 participants, when compared to year 7 participants, for both volume and intensity. This highlights the importance of physical activity for older adolescents, who are typically less active [61] and also subject to an age-related increase in blood pressure [62]. The present study also adds further novel insights with regards to the relationship between the activity volume metric and IL-6 concentration, as well as the intensity gradient and IL-1β concentration. Interestingly, the negative association observed between average acceleration and IL-6 concentration, as well as intensity gradient and IL-1β concentration, was exclusive to year 7 participants. The present study is the first to report that these relationships may be moderated by age across adolescence and thus future research should aim to replicate such findings and also examine the potential mechanism responsible.

The present study is also the first to examine the associations between the newly proposed physical activity metrics, average acceleration and intensity gradient, and cognitive function in adolescents. These data from the present study did not suggest there were any associations between average acceleration and intensity gradient with cognitive function. Whilst previous studies have reported that device-measured physical activity is beneficially associated with measures of cognitive function [16, 17], others do not [18]. Discrepancies amongst these findings may be explained by the different processing techniques used, as well as the summary metrics used to represent physical activity. Indeed, previous work focused solely on moderate-to-vigorous physical activity from self-report measures [17], whereas the present study utilised novel metrics that consider the whole physical activity intensity spectrum [22]. Despite the use of these new metrics, the present study may be limited by a small sample size (particularly for older adolescents; 14–15 y). Indeed, more work using these metrics with much larger samples is required to fully establish the nature of relationship between physical activity and cognition in adolescents.

It has been argued that physical fitness, as a state (which to some extent reflects chronic physical activity), is a better measure to use than physical activity itself which varies greatly from day-to-day, when examining the relationship with cognitive function [18]. This is supported by evidence showing that the associations between moderate-to-vigorous physical activity and cognitive function do not hold when physical fitness was accounted for [16]. This suggests that it is physical fitness, rather than physical activity per se, which is important for cognitive function and only some physical activities, of a sufficient intensity to enhance physical fitness, will contribute to enhanced cognition. Indeed, when considering physical fitness, physical activity and adiposity (waist circumference), the findings of the present study only found associations between physical fitness and cognitive performance, suggesting that interventions to enhance cognition in adolescents should focus on improving physical fitness, rather than simply increasing physical activity, or reducing adiposity.

The present study found that adiposity was important for cardiometabolic health, whereby waist circumference was positively associated with IL-6 concentration, although interestingly this was exclusive to year 10 participants. The findings of the present study support the empirical findings of Dring et al. [28], whereby sum of skinfolds (also indicative of greater adiposity) was positively associated with IL-6 concentration. Indeed, it is generally recognised that adolescents who display greater adiposity have higher concentrations of inflammatory cytokines [63]. Waist circumference is a recommended proxy of visceral adipose tissue, which is known as an endocrinological tissue that secretes inflammatory mediators, particularly IL-6 [64]. It is thought that the adipose tissue-derived IL-6 is mechanistically involved in the development of insulin resistance [5] and subsequent occurrence of cardiometabolic diseases, such as cardiovascular disease and type 2 diabetes [65]; with the present study providing novel evidence of a positive relationship between waist circumference and IL-6 concentration, indicative of poorer cardiometabolic health.

Whilst the present study provides novel insight as previously discussed, it is not without limitations. Firstly, the present study is cross-sectional in nature which precludes any inference of causality in the observed associations. Furthermore, although a commonly used field-based measurement of physical fitness was used (MSFT), a different version (15 m shuttles) than what is typically used (20 m shuttles) in paediatric exercise research was used, due to facility constraints at participating schools. The present study also recruited fewer year 10 participants compared to year 7, which may limit the conclusions in these older adolescents. As a result of this, the models created were parsimonious in nature and did not account for other important independent variables. Indeed, future work utilising large samples should seek to replicate such findings whilst accounting for additional independent variables.

## Conclusions

In summary, the present study highlights that a higher physical fitness is associated with enhanced cardiometabolic health and cognitive function in adolescents. Furthermore, the present study highlights important associations between a higher volume and intensity of physical activity (assessed using novel metrics; average acceleration and intensity gradient) with reduced risk factors for cardiometabolic disease (blood pressure, IL-1β); although a further interesting observation was that these associations were stronger in year 10 participants than year 7 participants. This suggests that older adolescents (14–15 y) may require a more specific targeted approach to promote physical activity, to subsequently enhance cardiometabolic health and cognition. In addition, future longitudinal research is required to track cardiometabolic health and cognitive function across adolescence, and in particular to track the importance of changes in physical activity, physical fitness and adiposity for these key outcome measures.

## Data Availability

Data are available upon request from the corresponding author.

## Abbreviations

**BDNF**: Brain-derived Neurotrophic Factor
**BMI**: Body Mass Index
**DBP**: Diastolic Blood Pressure
**HOMA-IR**: Homeostatic Model Assessment of Insulin Resistance
**IL**: Interleukin
**MAP**: Mean Arterial Pressure
**MSFT**: Multi-stage Fitness Test
**MVPA**: Moderate-to-vigorous Physical Activity
**SBP**: Systolic Blood Pressure
